# Preceding psychological factors and calorie intake in patients with type 2 diabetes: investigation by ecological momentary assessment

**DOI:** 10.1186/s13030-019-0161-4

**Published:** 2019-09-04

**Authors:** Shuji Inada, Yoko Iizuka, Ken Ohashi, Hiroe Kikuchi, Yoshiharu Yamamoto, Takashi Kadowaki, Kazuhiro Yoshiuchi

**Affiliations:** 1 Department of Stress Sciences and Psychosomatic Medicine, Graduate School of Medicine, The University of Tokyo, 7-3-1 Hongo, Bunkyo-ku, Tokyo, Japan; 2 Department of Diabetes and Metabolic Diseases, Graduate School of Medicine, The University of Tokyo, 7-3-1 Hongo, Bunkyo-ku, Tokyo, Japan; 3 Department of Internal Medicine, National Cancer Center Hospital, 5-1-1 Tsukiji, Chuo-ku, Japan; 4 Department of Psychosomatic Medicine, Center Hospital, National Center for Global Health and Medicine, 1-21-1 Toyama, Shinjuku-ku, Tokyo, Japan; 5 Educational Physiology Laboratory, Graduate School of Education, The University of Tokyo, 7-3-1 Hongo, Bunkyo-ku, Tokyo, Japan; 6 Department of Diabetes and Metabolic Diseases and Department of Prevention of diabetes and lifestyle-related diseases, Graduate School of Medicine, The University of Tokyo, 7-3-1 Hongo, Bunkyo-ku, Tokyo, Japan

**Keywords:** Ecological momentary assessment, Type 2 diabetes, Electronic food diary, Psychological factors, Multilevel modeling

## Abstract

**Background:**

Psychological factors have been reported to have influence on the eating habits of patients with diabetes. However, previous studies have used questionnaires to investigate the association, and thus include recall bias. To overcome this disadvantage, ecological momentary assessment (EMA) can be used to record subjective symptoms and behavior in subjects’ daily lives. Therefore, the aim of the present study was to investigate the influence of preceding psychological factors on calorie intake using computerized EMA for 6 months.

**Methods:**

The participants were nine outpatients with type 2 diabetes, aged 34–72. They were instructed to use a personal digital assistant as an electronic diary for 6 months to record subjective symptoms, such as psychological stress, anxiety, and depressive mood, and the food and drink that they consumed. The association between a preceding psychological factor and calorie intake within 5 hours was investigated using multilevel modeling.

**Results:**

Preceding psychological stress was positively associated with calorie intake from snacks. Preceding psychological stress, anxiety, and depressive mood were negatively associated with calorie intake from regular meals.

**Conclusions:**

Preceding psychological factors influence the calorie intake of patients with type 2 diabetes. Understanding the role of these factors will be useful for developing psychological interventions to prevent overeating.

**Trial registration:**

The trial registration number: UMIN000002992. Date of registration: 2010/01/07.

## Background

Psychological factors, such as anxiety and depressive mood, have been reported to have influence on the eating habits of patients with life-style diseases, such as diabetes, which raises the risks of cardiovascular diseases [1]. Many studies have used questionnaires to investigate the association between psychological factors and eating habits, as in this previous study. However, the use of questionnaires is problematic because of their potential for recall bias [2]. To overcome this disadvantage, ecological momentary aqssessment (EMA), which avoids recall bias, has been used in the area of behavioral medicine to record subjective symptoms and behavior in subjects’ daily lives.

Some previous studies have been done to investigate the association between psychosocial factors and food intake using EMA [3–6]; however, these studies included methodological problems. First, most [3–5] used paper-and-pencil diaries to record subjective symptoms and food intake, while Tomiyama et al. [6] used an electronic diary (i.e. computerized EMA). Compared with EMA using paper-and-pencil diaries, computerized EMA has the advantage of avoiding ‘faked compliance’, i.e. disguised compliance by recording data at times other than those designated, by recording the time of data input [7]. In addition, the study by Tomiyama, which used computerized EMA, only assessed two days.

The aim of the present study was to do a long-term, quantitative investigation of the influence of preceding psychological factors on calorie intake, using computerized EMA for six months.

## Methods

### Participants and procedures

The participants were nine outpatients with type 2 diabetes diagnosed according to the current criteria of the Japan Diabetes Society [8] (three men; median age = 49 years, range = 34 to 72) who also took part in another study [9]. Inclusion criteria were age ≥ 20 and an outpatient with type 2 diabetes at the University of Tokyo Hospital. Participants were excluded if they had HbA1c ≥ 8.4% (corresponding to 8.0% before converting to the NGS*P* value, which was the criterion for poorly controlled type 2 diabetes), had any active mental disorder, followed a restrictive diet with protein ≤0.5 g/kg/day or salt ≤5 g/day due to another disease, had cognitive impairment, or had severe complications that interfered with self-care activities. After providing written informed consent, participants were instructed to record daily dietary intake using a PDA-based food diary, SELFOOD [10], and to record mood states, such as anxiety and depression, on a personal digital assistant (PDA) (WS020SH, Sharp Corp, Osaka, Japan, 135 g, 50x116x17.9 mm, Windows Mobile 6.1) that was equipped with a 3.0-in color liquid crystal display and a touch panel manipulated by a finger. They were asked to record as soon as possible after eating for 6 consecutive months. Anthropometric measurements, including height, body weight, and blood pressure, were performed at the beginning of the study. Clinical data (e.g. fasting plasma glucose level, hemoglobin A1c value, and duration of diabetes) were collected at their most recent visit with a health care provider. They visited their physician regularly, and continued to take the medications prescribed for their conditions during the study period.

### Dietary intake

We developed an electronic food diary, “SELFOOD”, for use in a personal digital assistant (PDA). Its accuracy has been confirmed by comparison with a 24-h recall interview by registered dietitians [10]. SELFOOD includes a reference database with 423 color photographs of food and drink items and allows patients to add their own meals and recipes to the database if their nutrient information is given. SELFOOD calculates energy intake after every input. Calorie intake was summed on an input-by-input basis and converted to a daily bar graph with a horizontal line indicating the target value of daily calorie intake.

### Momentary psychological stress and mood states

A PDA was used as electronic diary to record psychological stress, anxiety, and depressive mood [11]. The subjects were instructed on the use of the device and given manuals before the beginning of the study period. In addition, they practiced manipulating the device with the author until they became accustomed to its use.

The subjects used the PDAs for six consecutive months. Signal-contingent recordings were defined as recordings that were prompted with a beep as a signal [2], which was programmed to occur randomly within an interval of 30 mins, around 10:00 and 15:00. If the subjects did not enter a recording when the computer beeped, they were allowed to postpone input for 30 min. Recordings not made within 30 min were cancelled. The subjects were also asked to record their psychological stress, anxiety, and depressive mood when they woke up and when they went to bed by choosing “waking up” or “going to bed” from the menu. After selecting a “going to bed” recording, to avoid sleep disturbance the computers suspended the signal-contingent recordings until a selected “wake up” recording was played. Signal-contingent recordings and recordings when waking up and going to bed were treated as scheduled recordings. Momentary psychological stress, anxiety, and depressive mood were rated with a visual analog scale (VAS) that ranged from 0 to 100, which was displayed on the screen.

### Statistical analysis

Multilevel modeling was used for statistical analyses to investigate preceding psychological factors on dietary intake because the dataset in this study had a nested structure in which a number of recordings belonged to each participant. Pairs of preceding psychological factors and dietary intake were lagged in the range of − 5 to 0 h [12]. The level of significance was set at 0.05. SAS Proc Mixed (SAS 9.3, SAS Institute Inc., Cary, NC, USA) was used. Each dietary intake recording was paired with the preceding momentary recording of psychological stress and mood states. Dietary intake was treated as the dependent variable. Momentary psychological stress, anxiety, and depressive mood were separately modeled as the independent variable, either as a fixed or random effect. Preceding momentary recordings of psychological factors were recorded within five hours before dietary intake, and there was no dietary intake between a preceding momentary recording of psychological factors and the dependent variable of dietary intake. Using these stratified time lags, models with different combinations of independent variables (with/without a preceding psychological factor as a fixed/random effect) were tested. Goodness of fit was compared to determine the best-fit model with a − 2 log likelihood function and χ^2^ test when one model was nested in the other; otherwise, Akaike’s Information Criterion (AIC) was used. The level-1 (within-individual level) intercept was modeled as a random effect. In all analyses, the variance-covariance matrix (G matrix) was modeled as unstructured.

## Results

### Subject characteristics

The characteristics of subjects are shown in Table 1.

**Table 1:** Subject characteristics

Number (male/female)	9 (6/3)
Age (years)	49 (34,72)
BMI (kg/m^2^)	25.5 (20.3,52.3)
Duration of illness (years)	6 (0.5, 16)
Blood HbA1c (%)	7.4 (6.2,8.2)
Medication (n)	
Oral hypoglycemic agents	9
Oral hypoglycemic agents + insulin	4

Values are given as median (minimum, maximum) unless otherwise stated

### Recording profiles

In total, the data from 1353 days of recordings was available for analysis. The compliance rate for regular dietary intake was 77.2%, while the compliance rate for signal-contingent recordings of psychological factors was 42.8%. With regard to pairing dietary intake and preceding psychological factors, 1517 of 3667 dietary intakes could be paired with preceding psychological factors.

### Preceding psychological stress and dietary calorie intake

Preceding psychological stress was significantly, negatively associated with dietary intake for lunch and dinner, but positively associated for snacks (Tables 2, 3 and 4). The final model is as follows:

**Table 2:** Effect of preceding psychological stress on calorie intake, for lunch

Lunch	Coefficient (S.E.)	*F* value	*P* value
Intercept (γ00)	565.71 (47.45)		< 0.0001
Accompanying person (γ10)		F(1,7) = 19.04	0.0033
Alone	446.84 (35.59)		
Someone	525.91 (36.04)		
Place (γ20)		F(3,10) = 11.39	0.0015
Home	489.18 (34.57)^a^		
Office/school	410.24 (41.90)^a^		
Dining out	565.57 (35.39)		
Other	480.52 (45.09)		
Stress (γ30)	−1.7259 (0.4369)	F(1,467) = 15.61	< 0.0001

^a^Significant difference with “Dining out” (*p* < 0.05 for all using Tukey-Kramer’s correction)

**Table 3:** Effect of preceding psychological stress on calorie intake, for dinner

Dinner	Coefficient (S.E.)	*F* value	*P* value
Intercept (γ00)	− 256.15(297.61)		0.4179
Accompanying person (γ10)		F(1,6) = 5.58	0.0562
Alone	484.64(40.44)		
Someone	548.53(37.51)		
Place (γ20)		F(3,4) = 12.26	0.0174
Home	577.45(30.41)		
Office/school	366.44(57.04)^*#^		
Dining out	684.19(44.51)		
Other	438.27(60.66)^*^		
Stress (γ30)	−1.2209(0.5118)	F(1,385) = 12.26	0.0175
Target calorie (γ01)	0.5106(0.1881)	F(1,7) = 7.37	0.0300

^*^Significant difference with “Dining out” (*p* < 0.05 for all using Tukey-Kramer’s correction)

^#^Significant difference with “Home” (*p* < 0.05 for all using Tukey-Kramer’s correction)

**Table 4:** Effect of preceding psychological stress on calorie intake, for snacks

Snacks	Coefficient (S.E.)	*F* value	*P* value
Intercept (γ00)	93.54(48.68)		0.0961
Accompanying person (γ10)		F(1,5) = 7.36	0.0422
Alone	100.34(41.84)		
Someone	154.91(41.99)		
Place (γ20)		F(3,7) = 6.62	0.0188
Home	158.14(41.11)		
Office/school	69.24(43.62)^*#^		
Dining out	189.18(49.57)		
Other	93.93(44.07)^#^		
Stress (γ30)	1.2513(0.5403)	F(1,186) = 5.36	0.0216

^*^Significant difference with “Dining out” (*p* < 0.05 for all using Tukey-Kramer’s correction)

^#^Significant difference with “Home” (*p* < 0.05 for all using Tukey-Kramer’s correction)

Level 1 equation:

$$ {\mathrm{Y}}_{\mathrm{ij}}={\uppi}_{0\mathrm{i}}+{\uppi}_{1\mathrm{i}}{\mathrm{Company}}_{\mathrm{ij}}+{\uppi}_{2\mathrm{i}}{\mathrm{Place}}_{\mathrm{ij}}+{\uppi}_{3\mathrm{i}}{\mathrm{Stress}}_{\mathrm{ij}}+{\upvarepsilon}_{\mathrm{ij}} $$

Level 2 equation:

$$ {\displaystyle \begin{array}{l}{\pi}_{0\mathrm{i}}={\gamma}_{00}+{\zeta}_{0\mathrm{i}}\left({\pi}_{0\mathrm{i}}={\gamma}_{00}++{\gamma}_{01}{\mathrm{TargetCal}}_{\mathrm{i}}+{\zeta}_{0\mathrm{i}}\mathrm{for}\ \mathrm{dinner}\right)\\ {}{\pi}_{1\mathrm{i}}={\gamma}_{10}\\ {}{\pi}_{2\mathrm{i}}={\gamma}_{20}\\ {}{\pi}_{3\mathrm{i}}={\gamma}_{30}\end{array}} $$

where Y_ij_ is each momentary calorie intake for the ith patient; Company_ij_ is a corresponding accompanying person, recorded as alone or with someone; Place_ij_ is the corresponding eating place; home, office/school, dining out, or other (e.g. outdoors); Stress_ij_ is the corresponding preceding psychological stress; TargetCal_i_ is the target calorie intake for the ith patient. γ_00_ is the average true value of mean calorie intake when all predictors are zero. π_1i_, π_2i_, and π_3i_ are the individual i’s slopes representing the effect of an accompanying person(s), eating place(s), or preceding psychological stress on momentary calorie intake, respectively, and γ_10_, γ_20_, and γ_30_ are the average slopes. ε_ij_ and ζ_0i_ are residuals at each level. Including ζ_0i_ in the equation means that the intercept was modeled as random, which suggests that intercept can vary across individuals. The second level 2 equation included no residual, which means that the effect of an accompanying person(s), eating place(s), or preceding psychological stress on momentary calorie intake was modeled as a fixed effect.

### Preceding anxiety and dietary calorie intake

Preceding anxiety was significantly, negatively associated with dietary intake only for breakfast (Table 5). The final model is as follows:

**Table 5:** Effect of preceding anxiety on calorie intake, for breakfast

Breakfast	Coefficient (S.E.)	*F* value	*P* value
Intercept (γ00)	596.20(50.58)		<0.0001
Accompanying person (γ10)		F(1,4) = 7.68	0.0502
Alone	418.98(35.84)		
Someone	489.73(37.17)		
Place (γ20)		F(3,5) = 4.45	0.0708
Home	413.79(29.72)		
Office/school	375.90(66.19)		
Dining out	516.49(49.69)		
Other	511.26(51.49)		
Anxiety (γ30)	−1.8047(0.7633)	F(1,395) = 5.59	0.0185

Level 1 equation:

$$ {\mathrm{Y}}_{\mathrm{ij}}={\uppi}_{0\mathrm{i}}+{\uppi}_{1\mathrm{i}}{\mathrm{Company}}_{\mathrm{ij}}+{\uppi}_{2\mathrm{i}}{\mathrm{Place}}_{\mathrm{ij}}+{\uppi}_{3\mathrm{i}}{\mathrm{Anxiety}}_{\mathrm{ij}}+{\upvarepsilon}_{\mathrm{ij}} $$

Level 2 equation:

$$ {\displaystyle \begin{array}{l}{\pi}_{0\mathrm{i}}={\gamma}_{00}+{\zeta}_{0\mathrm{i}}\\ {}{\pi}_{1\mathrm{i}}={\gamma}_{10}\\ {}{\pi}_{2\mathrm{i}}={\gamma}_{20}\\ {}{\pi}_{3\mathrm{i}}={\gamma}_{30}\end{array}} $$

where Y_ij_ is each momentary calorie intake for the ith patient; Company_ij_ is a corresponding accompanying person, either alone or with someone; Place_ij_ is the corresponding eating place, home, office/school, dining out, or other; Anxiety_ij_ is the corresponding preceding anxiety.

### Preceding depressive mood and dietary calorie intake

Preceding depressive mood was significantly, negatively associated with dietary intake only for lunch (Table 6). The final model is as follows:

**Table 6:** Effect of preceding depressive mood on calorie intake, for lunch

Lunch	Coefficient (S.E.)	*F* value	*P* value
Intercept (γ00)	564.61(43.27)		<0.0001
Accompanying person (γ10)		F(1,7) = 22.26	0.0022
Alone	442.80(29.85)		
Someone	528.15(30.60)		
Place (γ20)		F(3,10) = 11.70	0.0013
Home	491.43(28.81)^*^		
Office/school	414.13(37.15)^*^		
Dining out	569.16(29.91)		
Other	467.18(40.71)		
Depressive mood (γ30)	−3.6393(0.9826)	F(1,467) = 13.72	0.0002

^*^Significant difference with “Dining out” (*p* < 0.05 for all using Tukey-Kramer’s correction)

Level 1 equation:

$$ {\mathrm{Y}}_{\mathrm{ij}}={\uppi}_{0\mathrm{i}}+{\uppi}_{1\mathrm{i}}{\mathrm{Company}}_{\mathrm{ij}}+{\uppi}_{2\mathrm{i}}{\mathrm{Place}}_{\mathrm{ij}}+{\uppi}_{3\mathrm{i}}{\mathrm{Depression}}_{\mathrm{ij}}+{\upvarepsilon}_{\mathrm{ij}} $$

Level 2 equation:

$$ {\displaystyle \begin{array}{l}{\pi}_{0\mathrm{i}}={\gamma}_{00}+{\zeta}_{0\mathrm{i}}\\ {}{\pi}_{1\mathrm{i}}={\gamma}_{10}\\ {}{\pi}_{2\mathrm{i}}={\gamma}_{20}\\ {}{\pi}_{3\mathrm{i}}={\gamma}_{30}\end{array}} $$

where Y_ij_ is each momentary calorie intake for the ith patient; Company_ij_ is a corresponding accompanying person, either alone or with someone; Place_ij_ is the corresponding eating place, home, office/school, dining out, or other; Depression_ij_ is the corresponding preceding depressive mood.

## Discussion

Using computerized EMA for 6 months, significant within-individual relationships between preceding psychological factors and calorie intake within 5 hours were shown in patients with type 2 diabetes. Although a causal relation could not be established, our results support the possibility that psychological factors can influence the calorie intake of patients with type 2 diabetes, which has not been investigated previously on a daily-life basis.

In the present study, the results showed differences in the association between preceding psychological factors and calorie intake among types of food. With regard to snacks, we showed that preceding psychological stress was positively associated with calorie intake. This result is consistent with the results of previous studies [3, 5, 6]. In the study by Lowe et al. [3], calorie intake from snacks increased when subjects with obesity were in a negative mood, although the study did not investigate the relation proportionally between negative mood and calorie intake. O’Connor et al. [5] reported a positive association between daily hassles and calorie intake from snacks, using only end-of-day assessments that did not include momentary psychological factors. Tomiyama et al. [6] used computerized EMA in a short two-day report that found preceding negative mood to be positively associated with calorie intake of snacks. Because the results of studies using such different methods were consistent, psychological stress would seem to increase calorie intake of snacks. Eating snacks may be a coping mechanism for reducing psychological stress [13].

With regard to regular meals, preceding psychological stress, anxiety, and depressive mood were negatively associated with calorie intake. Negative mood may cause not only emotional eating, but appetite loss mediated by autonomic reaction. Because the patients were following diet therapy advice, the calorie intake in their regular meals might have been affected by a physical reaction rather than by an emotional reaction. The results of previous studies were not consistent regarding the association between psychological factors and calorie intake. Lowe et al. [3] found no association between negative mood and the calorie intake from regular meals, while Patel et al. [4] reported that the calorie intake from regular meals was greater for “negative mood” than for “neutral mood”. Because all of the subjects were women in the two previous studies, it would be difficult to compare them to the results of the present study. Therefore, future studies will necessary to confirm the association between preceding psychological factors and regular meals.

When controlling for psychological factors, calorie intake was generally greater when eating with than without another person(s) and when eating out than when eating at home. This result supports the results shown in a previous study that calorie intake increased in social situations [4].

There were some limitations in the present study. First, the sample size was small. Second, most of the patients in the present study were men. Therefore, future studies with more patients including more men and women will be necessary. Also, there was no control group of healthy people, which makes it impossible to expand the results in the present study to healthy people. In addition, some of the dietary intake could be paired with preceding psychological factors, which might be subject to selection bias.

## Conclusions

Preceding psychological factors influence the calorie intake of patients with type 2 diabetes. Better understanding these factors will be helpful for developing psychological interventions to prevent overeating.

## Data Availability

We are not able to share our data because sharing data is not permitted by our hospital or the ethics committee.

## Abbreviations

AIC: Akaike’s information criterion
EMA: ecological momentary assessment
HbA1c: hemoglobin A1c
PDA: portable digital assistant
VAS: visual analog scale
